# Evaluation of the GlideScope Direct: A New Video Laryngoscope for Teaching Direct Laryngoscopy

**DOI:** 10.1155/2012/820961

**Published:** 2012-06-24

**Authors:** Darwin Viernes, Allan J. Goldman, Richard E. Galgon, Aaron M. Joffe

**Affiliations:** ^1^Department of Anesthesiology and Pain Medicine, University of Washington, 325 Ninth Avenue, Box 359724, Seattle, WA 98104, USA; ^2^Department of Anesthesiology, Swedish Medical Center, Seattle, WA 98104, USA; ^3^Department of Anesthesiology, University of Wisconsin Hospital and Clinics, Madison, WI 53792, USA

## Abstract

*Background*. Teaching direct laryngoscopy is limited by the inability of the instructor to simultaneously view the airway with the laryngoscopist. Our primary aim is to report our initial use of the GlideScope Direct, a video-enabled, Macintosh laryngoscope intended primarily as a training tool in direct laryngoscopy. *Methods*. The GlideScope Direct was made available to anyone who planned on performing direct laryngoscopy as the primary technique for intubation. Novices were those who had performed <30 intubations. *Results*. The GlideScope Direct was used 123 times as primarily a direct laryngoscope while the instructor viewed the intubation on the monitor. It was highly successful as a direct laryngoscope (93% success). Salvage by indirect laryngoscopy occurred in 7/9 remaining patients without changing equipment. Novices performed 28 intubations (overall success rate of 79%). In 6 patients, the instructor took over and successfully intubated the patient. Instructors used the video images to guide the operator in 16 (57%) of those patients. Seven different instructors supervised the 28 novices, all of who subjectively felt advantaged by having the laryngoscopic view available. *Conclusions*. The GlideScope Direct functions similarly to a Macintosh laryngoscope and provides the instructor subjective reassurance, while providing the ability to guide the trainee laryngoscopist.

## 1. Introduction

Many experts predict that video laryngoscopy (VL) will eventually replace direct laryngoscopy (DL) as the primary laryngoscopic technique when attempting tracheal intubation. However, recent studies comparing the GlideScope video laryngoscope (Verathon, Bothell, WA, USA) and the Pentax AWS (Ambu, Inc., Glen Burnie, MD, USA) to Macintosh DL for intubating morbidly obese subjects failed to support the superiority of VL in this patient population [1, 2]. Additionally, financial constraints, particularly in developing nations, make substitution of the far more costly VL devices for the traditional DL blades impractical. Thus, for the foreseeable future, DL will remain an essential skill for health care providers responsible for tracheal intubation [3]. 

The restricted ability to share a trainee's view of the patient's airway with the instructor is a significant limitation when teaching DL. The traditional instructor/trainee relationship involves blinded verbal feedback to the trainee and/or the instructor “looking over the shoulder” to share the view of the airway. In an attempt to improve the quality of trainee education and patient safety, airway educators have developed methods to share and expand the view of the airway. These methods have included the replacement of the laryngoscope light bulb with a fiberoptic bronchoscope, a head mounted video system (Airway Cam), or the use of indirect rigid video laryngoscopes [4–6].

**Figure 1:**
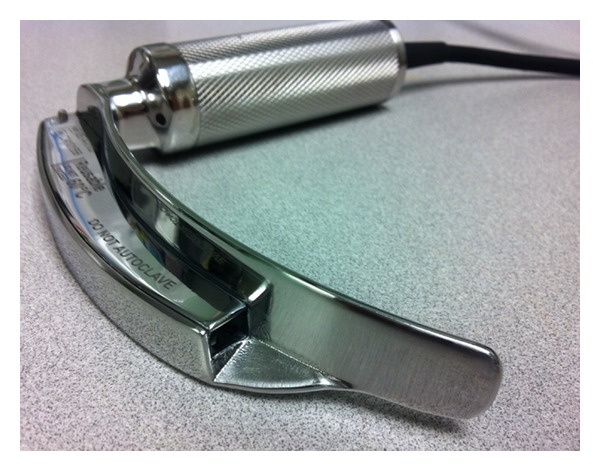
GlideScope Direct video laryngoscope. The video camera and light source are embedded where a light bulb is located on a traditional Macintosh laryngoscope.

The GlideScope Direct (GSD, Verathon, Bothell, WA, USA) is a newer, reusable video-enabled Macintosh laryngoscope marketed for the specific purpose of teaching direct laryngoscopy. In contradistinction to other devices in the GlideScope product line, such as the GVL, Cobalt, and Ranger, which have a view axis of roughly 290°, the GSD is a Macintosh 3.5 blade with a traditional view axis of 90° along the operator's direct line of sight [7]. The camera and light source are located in the same position as the light source on the standard Macintosh blade thus providing a view angle of up to 290° to the instructor [7] and are fully enclosed within the handle-blade assembly similar to the original GVL device. The power input and video output are carried via a cord permanently integrated into the top of the handle, which interfaces with the GlideScope Advanced Video Laryngoscope (AVL) monitor (Figure 1). The AVL monitor provides color, digital quality picture with integrated, one-touch, real-time recording with flash memory capability to store up to one hour of continuous video. Stored video may be transferred to an external storage device via an integrated USB port for playback and review. Reports of its use in the clinical arena are currently lacking, however. As such, the purpose of this prospective, observational study was to describe our initial experience using the GSD with particular attention to its use as a teaching tool during routine airway management.

## 2. Methods

This study was approved by the University of Washington Minimal Risk Institutional Review Board (Seattle, WA) without the need for informed consent, and conducted from March 1, 2011 through April 1, 2011 at the Harborview Medical Center (HMC, Seattle, WA), a 413-bed municipal medical center affiliated with the University of Washington, having 28 dedicated operating rooms, staffed by attending anesthesiologists, either working solo or supervising anesthesiology trainees and/or nurse anesthetists. Additionally, the operating rooms are a primary airway management training location for a number of nonanesthesia trained providers, including local emergency medical services personnel, flight nurses, and emergency and internal medicine trainees. 

The GSD was made available for any case for which the primary anesthesia team was planning to use direct laryngoscopy with a Macintosh blade as the initial technique for tracheal intubation. Patient selection, therefore, was nonrandom and only of convenience. Once the primary team requested the GSD, one of the authors (DV, AMJ) made themselves available to briefly explain the design and functionality of the device. The primary laryngoscopist was instructed to perform direct laryngoscopy as usual using the GSD and described pertinent airway structures as the blade was advanced into the airway. The AVL monitor was kept turned to the attending anesthesiologist at all times, unless the described CL grade by the primary laryngoscopist was >3. Under these conditions and at the discretion of the attending anesthesiologist, the laryngoscopist was given access to the AVL monitor view, and indirect laryngoscopy could be attempted. Alternatively, another technique, such as use of the GlideScope GVL, could be performed.

As per routine practice, all patients were brought to the operating room after intravenous access was achieved and standard monitors, including continuous surface electrocardiography, pulse oximetry, and an automated blood pressure cuff, were applied. The patient was preoxygenated with 100% oxygen and general anesthesia was instituted with standard intravenous induction drugs and dosages given by the attending anesthesiologist. All intubation attempts were then performed under general anesthesia. A second spare blade was available to overbridge time for sterilization after use, which was performed using a Steris System 1 tabletop machine per manufacturer recommendations.

Data was collected by one of the study authors in an anonymous format using a paper-based data collection form contemporaneously with airway management. Medical records were not accessed directly at any time. Demographic and airway exam variables collected included age, gender, height (cm), weight (kg), body mass index (kg·m^−2^), ASA physical status, Mallampati class, interincisor distance, thyromental distance, cervical mobility, and upper lip bite test. Trainee type and experience level, including prior intubation experience and number of intubation attempts, CL grade as reported by the primary laryngoscopist, and subjective assessment of overall airway management difficulty were also recorded. Additional, data collected during laryngoscopy included, conversion to instructor level airway management, CL grade as observed on the video monitor, and any remarks that instructors or trainees had about the device.

Data were entered into an electronic spreadsheet (Excel, Microsoft, Redmond, WA) and descriptively expressed as number (%) unless otherwise noted. For the purposes of analysis, a novice laryngoscopist was one who had performed <30 direct laryngoscopies, while an experienced laryngoscopist was considered one who had performed ≥30 laryngoscopies. Reinsertion of the blade of the device into the mouth or reinsertion of the tracheal tube into the mouth once it had been viewed on the video screen defined an “attempt” at direct and indirect laryngoscopy, respectively. Risk factors for difficult laryngoscopy and subsequent intubation were defined by the presence of one or more of the following: a Mallampati class >3 airway, thyromental distance <6 cm, interincisor gap <3 cm, reduced cervical spine motion, an inability to bite any part of upper lip, or history of difficult intubation documented by a prior anesthetic record or by patient self-report.

## 3. Results 

During the study period, 123 patients were intubated using the GSD. Patient and primary laryngoscopist characteristics are given in Tables 1 and 2. Overall intubation success using the GSD was 98% (121/123). First, second, and third intubation attempt success rates using the GSD for direct laryngoscopy were 87% (99/114), 12% (14/114), and 1% (1/114), respectively. In 9 cases, direct laryngoscopy was difficult and the laryngoscopist converted to using the video screen to perform indirect laryngoscopy, which proved successful in 7 of the 9 cases (77%) within 2 attempts. Two patients (1.6%) could not be intubated using the GSD, regardless of the approach (direct or indirect). These patients were successfully intubated using the GlideScope GVL. A summary of all 123 intubations is provided in Figure 3. Twenty-eight patients were intubated by novices with first and second attempt intubation success rates of 61% (17/28) and 18% (5/28), respectively. Six (21%) intubations were completed by the attending anesthesiologist. In comparison, experienced operators had first and second attempt direct laryngoscopy intubation success rates of 86% (82/95) and 8% (8/95), respectively. The instructor used the video images to aid the novice laryngoscopist 57% (16/28) of the time.

When the CL grade was described as II (*n* = 17) by the primary laryngoscopist, the view on the AVL screen was a CL-I grade in 16 (94%) cases and CL-II grade (no difference) in 1 (6%) case. When the primary laryngoscopist described a CL-III score (*n* = 9), the view on the AVL screen was a CL-I grade in 7 (78%) cases, CL-II grade in 1 (11%) case, and CL-III grade in 1 (11%) case. Finally, when the primary laryngoscopist described a CL-IV score (*n* = 2), the view on the AVL screen was a CL-II grade in both (100%) cases. There were no monitor views that were worse compared to the primary laryngoscopist's graded DL view.

## 4. Conclusions

**Figure 2:**
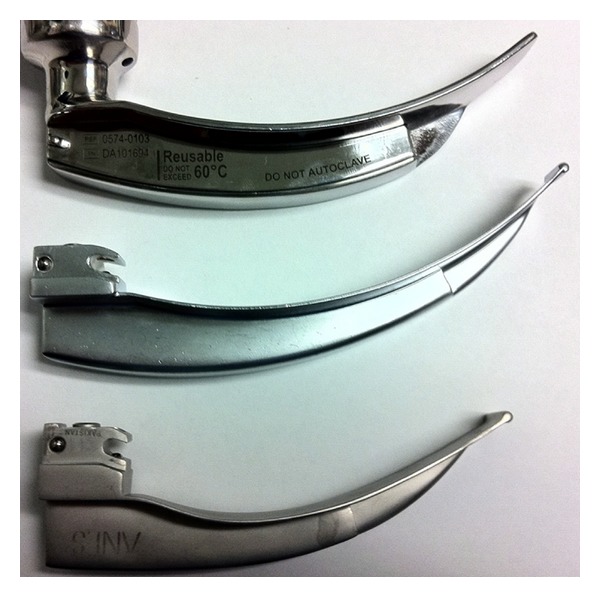
From top to bottom, comparison of the GlideScope Direct blade (top), Macintosh size 4 blade (middle), and a Macintosh size 3 blade (bottom).

**Figure 3:**
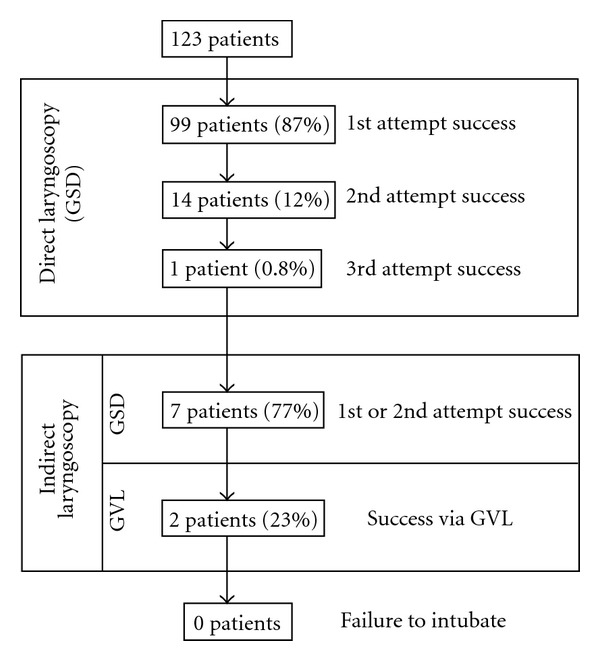
Description of airway management in patients using the GlideScope Direct.

**Figure 4:**
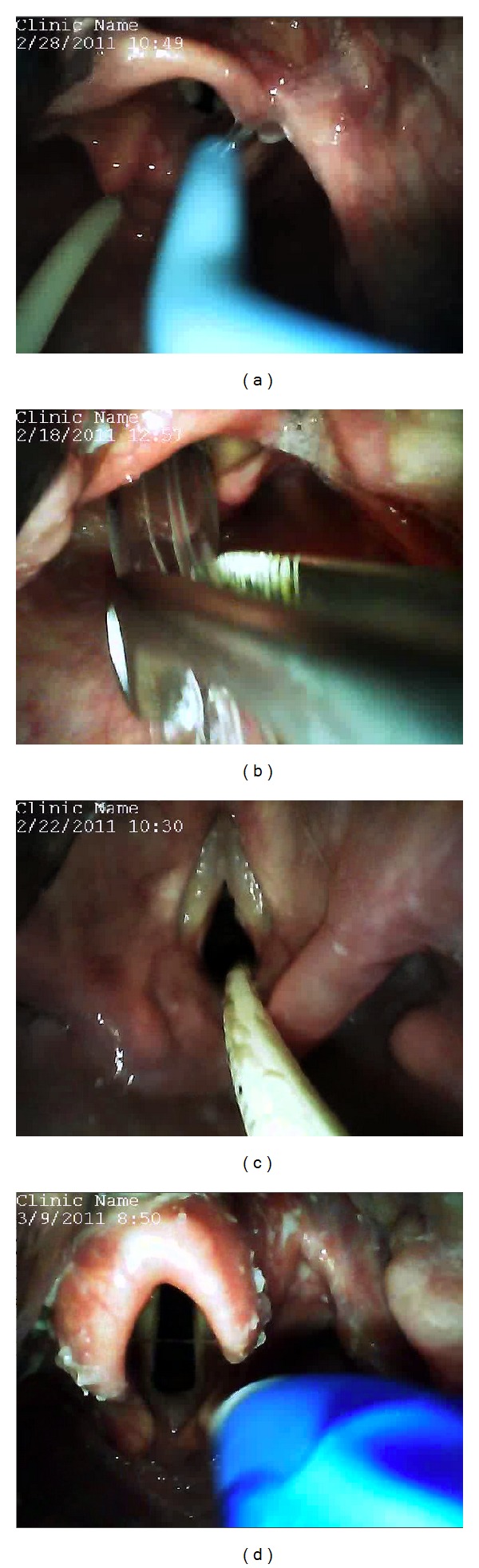
Examples of unique situations where the GlideScope Direct was found to be useful. (a) Endotracheal intubation with the aid of a bougie. (b) Nasotracheal Intubation with the use of Magill forceps. (c) Endotracheal tube exchange with the a Cook Airway Exchange Catheter. (d) Double-lumen endotracheal tube placement.

The main finding of our study is that the GSD can be used for direct laryngoscopy and tracheal intubation with a high success rate in patients without predictors of difficult intubation by both novice and experienced laryngoscopists, while its integrated video view provides an instructor with information to help guide tracheal intubation, which was utilized in a majority of cases. Subjectively, our users considered the GSD to have a similar feel to our standard direct laryngoscope handle and blade. It's light source allowed adequate visualization of airway structures without undue glare or reflection and attending anesthesiologists commented that they felt “comforted” by having the ability to share the airway view using the monitor. The instructors who were able to provide direct video feedback to trainees also noted single touch video recording to be helpful. Due to construction of the GSD blade being similar to that of a Macintosh blade (Figure 2), there are no limitations to the type of tube used (endotracheal tube, nasotracheal tube, double lumen tube, etc.) or intubation adjuncts (Magill forceps, bougie, airway exchange catheters, etc.) (Figure 4). 

The GSD is not the first device to be described as a video laryngoscope similar in construction to that of a Macintosh blade. Kaplan et al. first described the use of a “Video Macintosh Intubating Laryngoscope System (VMS)” in 235 patients [8] using a C-MAC video laryngoscope. Subsequently, there have been several studies demonstrating the use of the C-MAC video laryngoscope as a teaching device for novice intubators [9–11]. We noted that our novice intubators had direct laryngoscopy intubation success rates similar to previous reports [9–11]. 

In this study, we found that the CL grades obtained by the camera were slightly better than that obtained by the direct laryngoscopist. A recent review of airway devices by Levitan et al. noted that the proximal location (closer to the tip of the blade) of the video source in relation to the blade favorably influences the viewing angle [7]. In turn, the view displayed on the monitor may be better than that of the operator, which may falsely reassure the instructor. Fortunately, the GSD design allows easy conversion from direct to indirect laryngoscopy, which was necessary in 7 patients in our series, without changing equipment.

**Figure 5:**
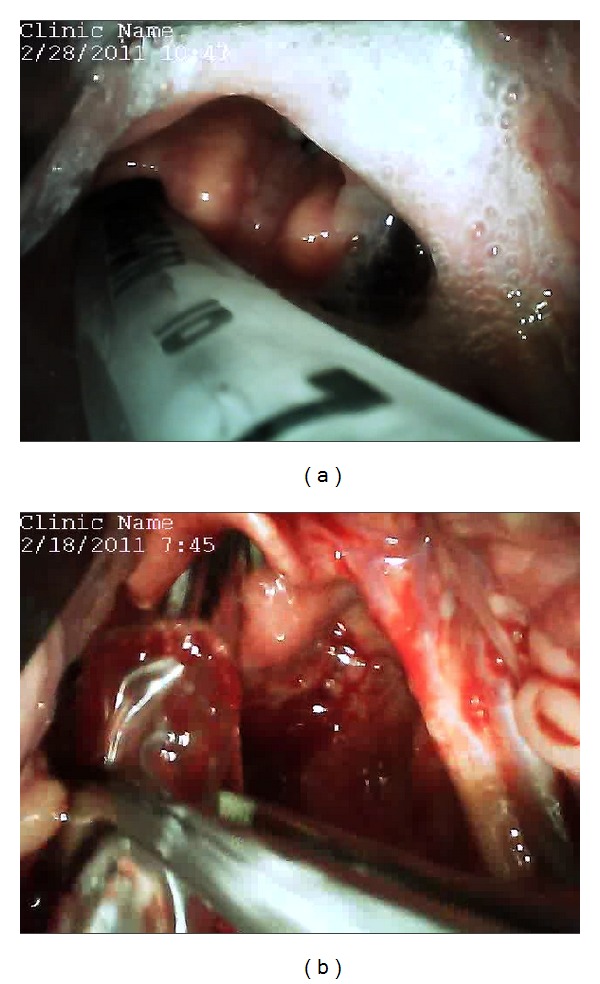
Examples of operator errors observed using the GlideScope Direct. (a) Esophageal intubation. (b) Grasping of the nasoendotracheal tube cuff with the Magill forceps.

Coincidentally, we were able to observe two instances of operator errors on the GVL monitor (Figure 5). These included an esophageal intubation and the grasping of the endotracheal tube cuff with Magill forceps. Further study is needed to evaluate the GSD's ability to observe these errors.

In summary, direct laryngoscopy and tracheal intubation using the GlideScope Direct is highly successful in patients without predictors of difficult intubation, when performed by both novice and experienced laryngoscopists, while its video feed proved useful to instructors for providing real-time guidance to trainees. Further study is needed to evaluate whether the GSD may influence the learning curve for novice users and if laryngoscopists are highly successful using a standard Macintosh blade after training with the GSD.

## Figures and Tables

**Table 1:** Patient characteristics. Data as %, *n* (%), or mean ± SD.

Age, yrs	46 ± 15
Sex, male	64
BMI, kg/m^2^	29 ± 7
Physical class	
1	25 (20)
2	48 (39)
3	49 (40)
4	1 (1)
Mallampati score ≥3	13 (11)
Thyromental distance <6 cm	26 (21)
Interincisor gap <3 cm	7 (6)
Reduced cervical mobility	15 (12)
Cannot bite any part of upper lip	6 (5)
History of difficult intubation	2 (2)
>1 risk factor for difficult laryngoscopy	13 (11)

BMI: body mass index.

**Table 2:** Number of intubations performed by prior training and intubation experience.

Training level	No.
Medical student	1
Paramedic/flight nurse	22
PGY-1	27
PGY-2	14
PGY-3	4
PGY-4	12
Nurse anesthetist	30
Staff	10
Other (nonanesthesia-based fellow)	3
Prior intubation experience	
0–10	5
11–30	23
31–50	5
51–100	8
>100	82
